# Supplementary material for: Short Linear Motifs Characterizing Snake Venom and Mammalian Phospholipases A2
Source: Toxins (Basel). 2021 Apr 20;13(4):290. doi: 10.3390/toxins13040290 (PMC8073766; doi:10.3390/toxins13040290)
Supplement: Supplementary file 1 [file toxins-13-00290-s001.zip › toxins-1156466 - supplementary .pdf]

Supplementary Materials: Short Linear Motifs Characterizing Snake Venom and Mam-malian Phospholipases A2

Caterina Peggion and Fiorella Tonello

Table S1. SLiMs contained in snake venom group I PLA2 and in mammalian PLA2G1B. In brackets the number of proteins belonging to each group; column numbers indicate the number of proteins in the corresponding group that possess the SLiMs listed in the next column.

N°	PLA2G1B (10)	N°	G1 NeuroMyotoxins (7)	N°	G1 Neurotoxins (13)
10	DOC_WW_Pin1_4	7	DEG_Nend_UBRbox_3	12	DEG_Nend_UBRbox_3
10	LIG_FHA_2	7	LIG_FHA_2	12	DOC_WW_Pin1_4
10	LIG_IBAR_NPY_1	7	LIG_SH2_STAT5	12	LIG_FHA_2
10	LIG_SH2_STAP1	6	MOD_GlcNHglycan	12	LIG_SH2_STAT5
10	LIG_SH2_STAT5	5	DOC_WW_Pin1_4	12	MOD_ProDKin_1
10	MOD_GlcNHglycan	5	MOD_ProDKin_1	9	MOD_GlcNHglycan
10	MOD_GSK3_1	4	CLV_PCSK_PC1ET2_1	8	DOC_USP7_MATH_1
10	MOD_ProDKin_1	4	MOD_NEK2_2	8	LIG_14-3-3_CanoR_1
10	TRG_ENDOCYTIC_2	4	TRG_ENDOCYTIC_2	7	CLV_PCSK_PC1ET2_1
9	LIG_SH2_CRK	3	DOC_MAPK_MEF2A_6	7	MOD_GSK3_1
9	MOD_NEK2_1	3	LIG_SH2_NCK_1	7	MOD_OFUCOSY
8	LIG_BRCT_BRCA1_1	3	LIG_SH2_SRC	7	TRG_ENDOCYTIC_2
7	TRG_ER_diLys_1	3	LIG_SH3_3	6	CLV_PCSK_SKI1_1
6	LIG_SH2_GRB2like	3	MOD_Plk_4	6	LIG_PTB_Apo_2
6	MOD_N-GLC_2	3	MOD_LATS_1	6	LIG_PTB_Phospho_1
5	LIG_SH3_3	3	MOD_PKA_1	6	LIG_SH2_NCK_1
5	MOD_CK1_1	3	MOD_PKA_2	6	MOD_N-GLC_2
5	MOD_CK2_1	3	TRG_ER_diLys_1	6	MOD_PKA_1
4	DOC_USP7_UBL2_3	2	CLV_NRD_NRD_1	6	MOD_PKA_2
4	LIG_BIR_II_1	2	CLV_PCSK_SKI1_1	5	LIG_SH2_CRK
3	CLV_PCSK_SKI1_1	2	DOC_CYCLIN_RXL_1	5	MOD_Cter_Amidation
2	CLV_PCSK_PC1ET2_1	2	DOC_USP7_MATH_1	4	LIG_SH2_GRB2like
2	MOD_SUMO_rev_2	2	MOD_GSK3_1	4	LIG_SH3_3
1	CLV_C14_Caspase3-7	2	MOD_PIKK_1	4	LIG_TYR_ITIM
1	CLV_NRD_NRD_1	2	TRG_NLS_MonoExtN_4	4	MOD_NEK2_2
1	DOC_CKS1_1	1	CLV_C14_Caspase3-7	4	MOD_Plk_4
1	DOC_SPAK_OSR1_1	1	DOC_MAPK_gen_1	4	TRG_ER_diLys_1
1	LIG_14-3-3_CanoR_1	1	DOC_PP1_RVXF_1	3	CLV_NRD_NRD_1
1	LIG_AP2alpha_2	1	LIG_LIR_Gen_1	3	DOC_MAPK_MEF2A_6
1	LIG_LIR_Gen_1	1	LIG_PTB_Apo_2	3	LIG_14-3-3_CterR_2
1	LIG_PDZ_Class_3	1	LIG_PTB_Phospho_1	3	LIG_LIR_Gen_1
1	LIG_SH2_NCK_1	1	LIG_SH2_GRB2like	3	LIG_SH2_SRC
1	MOD_N-GLC_1	1	MOD_CDK_SPxxK_3	3	LIG_TYR_ITIM
		1	MOD_CK1_1	3	MOD_LATS_1
		1	MOD_OFUCOSY	3	MOD_SUMO_rev_2
		1	MOD_SUMO_rev_2	3	TRG_LysEnd_APsAcLL_1
		1	TRG_ER_diArg_1	2	DOC_AGCK_PIF_1
		1	TRG_NLS_MonoExtC_3	2	DOC_MAPK_gen_1
				2	DOC_USP7_UBL2_3

2	MOD_CK1_1
2	MOD_PIKK_1
2	TRG_NLS_MonoExtN_4
1	CLV_C14_Caspase3-7
1	DEG_APCC_DBOX_1
1	DOC_CYCLIN_RXL_1
1	LIG_FHA_1
1	LIG_HCF-1_HBM_1
1	LIG_Integrin_isoDGR_1
1	MOD_CDK_SPK_2
1	MOD_CK2_1
1	MOD_NEK2_1
1	TRG_ER_diArg_1

Table S2. SLiMs contained in snake venom group II PLA2 and in mammalian PLA2G2A. In brackets the number of proteins belonging to each group; column numbers report the number of proteins in the corresponding group that possess the SLiMs listed in the next column.

N°	PLA2G2A (10)	N°	Myotx Not D49 (24)	N°	Myotoxins D49 (14)	N°	Neu-myot NOT D49 (5)	N°	Neuromyot D49 (9)	N°	Neurotoxins (11)
10	DOC_WW_Pin1_4	24	MOD_PKA_1	14	TRG_ENDOCYTIC_2	5	DOC_USP7_UBL2_3	9	LIG_SH2_STAT5	11	LIG_SH2_STAT5
10	LIG_FHA_1	23	TRG_ENDOCYTIC_2	13	LIG_SH2_STAT5	5	LIG_PDZ_Class_3	9	TRG_ENDOCYTIC_2	11	TRG_ENDOCYTIC_2
10	MOD_CDK_SPK_2	23	LIG_SH2_CRK	12	MOD_NEK2_1	5	LIG_SH2_CRK	7	CLV_PCSK_SKI1_1	10	LIG_FHA_2
9	LIG_14-3-3_CanoR_1	23	LIG_PDZ_Class_3	12	LIG_FHA_2	5	MOD_NEK2_1	7	DOC_USP7_UBL2_3	10	LIG_PDZ_Class_3
9	LIG_FHA_2	23	DOC_USP7_UBL2_3	11	MOD_GSK3_1	5	TRG_ENDOCYTIC_2	7	MOD_NEK2_1	10	MOD_CK1_1
9	MOD_GSK3_1	23	CLV_PCSK_SKI1_1	10	LIG_SH2_CRK	4	CLV_PCSK_SKI1_1	6	LIG_SH2_CRK	10	MOD_NEK2_1
9	MOD_OFUCOSY	20	MOD_NEK2_1	10	CLV_PCSK_SKI1_1	4	MOD_PKA_1	6	MOD_CK1_1	9	DOC_USP7_UBL2_3
8	CLV_PCSK_PC1ET2_1	17	MOD_PKA_2	9	LIG_PDZ_Class_3	4	MOD_Plk_1	6	MOD_OFUCOSY	9	LIG_SH2_CRK
8	DOC_USP7_UBL2_3	17	LIG_FHA_2	8	MOD_GlcNHglycan	3	LIG_14-3-3_CanoR_1	5	LIG_14-3-3_CanoR_1	8	MOD_CK2_1
8	MOD_Plk_1	17	LIG_14-3-3_CanoR_1	8	MOD_CK2_1	3	LIG_SH2_STAT5	5	LIG_FHA_1	7	CLV_PCSK_SKI1_1
8	MOD_ProDKin_1	15	LIG_SH2_STAT5	8	LIG_Rb_LxCxE_1	3	LIG_SH3_4	5	LIG_FHA_2	7	MOD_OFUCOSY
7	LIG_BRCT_BRCA1_1	13	MOD_Plk_4	8	DOC_USP7_UBL2_3	3	LIG_TRFH_1	5	LIG_PDZ_Class_3	5	CLV_NRD_NRD_1

7	MOD_CK1_1	13	MOD_OFUCOSY	7	MOD_CK1_1	3	MOD_PKA_2	5	LIG_SH2_STAP1	5	DOC_USP7_MATH_1
7	MOD_CK2_1	12	MOD_GSK3_1	7	CLV_PCSK_PC1ET2_1	3	MOD_Plk_4	5	MOD_CK2_1	5	DOC_WW_Pin1_4
7	MOD_N-GLC_1	12	MOD_Cter_Amidation	6	TRG_NLS_MonoExtN_4	2	LIG_BIR_II_1	5	MOD_GSK3_1	5	LIG_BRCT_BRCA1_1
7	MOD_PKA_2	12	CLV_NRD_NRD_1	6	MOD_ProDKin_1	2	LIG_BRCT_BRCA1_1	5	MOD_PKA_2	5	MOD_CDK_SPK_2
6	CLV_PCSK_SKI1_1	10	LIG_SH3_4	6	MOD_CDK_SPxxK_3	2	LIG_FHA_2	4	DOC_WW_Pin1_4	5	MOD_GSK3_1
6	LIG_SH2_GRB2like	10	LIG_Rb_LxCxE_1	6	LIG_14-3-3_CanoR_1	2	LIG_LIR_Gen_1	4	MOD_CDK_SPK_2	5	MOD_PKA_1
4	DEG_Nend_UBRbox_2	9	MOD_Plk_1	6	DOC_WW_Pin1_4	2	LIG_Rb_LxCxE_1	4	MOD_ProDKin_1	5	MOD_ProDKin_1
4	LIG_SH2_STAP1	7	MOD_N-GLC_1	6	DOC_MAPK_gen_1	2	MOD_CK1_1	3	DEG_Nend_UBRbox_2	4	LIG_LIR_Gen_1
4	TRG_ENDOCYTIC_2	6	MOD_SUMO_rev_2	6	DEG_Nend_UBRbox_3	2	MOD_OFUCOSY	3	DEG_Nend_UBRbox_3	4	MOD_GlcNHglycan
3	LIG_SH2_STAT5	6	LIG_SH2_STAP1	6	CLV_NRD_NRD_1	1	CLV_C14_Caspase3-7	3	DOC_MAPK_gen_1	4	MOD_PKA_2
3	LIG_UBA3_1	5	MOD_ProDKin_1	5	LIG_SH2_STAP1	1	DEG_APCC_DBOX_1	3	LIG_LIR_Gen_1	4	MOD_Plk_4
3	MOD_GlcNHglycan	5	MOD_N-GLC_2	4	MOD_Plk_4	1	DOC_MAPK_gen_1	3	LIG_Rb_LxCxE_1	3	DOC_MAPK_gen_1
3	MOD_NEK2_1	5	MOD_CK2_1	4	MOD_CDK_SPK_2	1	DOC_MAPK_MEF2A_6	3	MOD_GlcNHglycan	3	LIG_14-3-3_CanoR_1
3	TRG_ER_diArg_1	5	LIG_TRFH_1	3	TRG_ER_diArg_1	1	DOC_USP7_MATH_1	3	MOD_Plk_1	3	LIG_SH2_NCK_1
2	DOC_PP1_RVXF_1	5	DOC_MAPK_gen_1	3	MOD_PKA_1	1	DOC_WW_Pin1_4	2	CLV_NRD_NRD_1	3	MOD_PKB_1
2	LIG_TRAF2_1	4	MOD_ProDKin_1	3	MOD_NEK2_2	1	LIG_deltaCOP1_diTrp_1	2	CLV_PCSK_PC7_1	3	TRG_ER_diArg_1
2	MOD_PIKK_1	4	MOD_NEK2_2	3	MOD_Cter_Amidation	1	LIG_SH2_NCK_1	2	LIG_BRCT_BRCA1_1	2	DEG_Nend_UBRbox_3
2	MOD_Plk_4	4	MOD_CK1_1	3	LIG_FHA_1	1	LIG_SH2_SRC	2	LIG_MLH1_MIPbox_1	2	LIG_FHA_1
2	MOD_SUMO_rev_2	4	DOC_WW_Pin1_4	3	LIG_BRCT_BRCA1_1	1	LIG_SH2_STAP1	2	LIG_PDZ_Class_2	2	LIG_SH2_STAP1

1	CLV_C14_Caspase3-7	3	TRG_NLS_MonoExtN_4	3	CLV_PCSK_PC7_1	1	MOD_CDK_SPK_2	2	LIG_Pex14_1	2	LIG_UBA3_1
1	LIG_Pex14_2	3	CLV_PCSK_PC1ET2_1	2	TRG_ER_diLys_1	1	MOD_CDK_SPxK_1	2	LIG_SUMO_SIM_anti_2	1	CLV_C14_Caspase3-7
1	LIG_SH2_STAT3	2	MOD_Plk_2-3	2	MOD_Plk_1	1	MOD_CDK_SPxxK_3	2	LIG_TYR_ITAM	1	CLV_PCSK_FUR_1
1	TRG_ER_diLys_1	2	MOD_CDK_SPK_2	2	LIG_WW_1	1	MOD_CK2_1	2	MOD_NEK2_2	1	CLV_PCSK_PC1ET2_1
1	TRG_NLS_Bipartite_1	2	LIG_LIR_Gen_1	2	LIG_PDZ_Class_2	1	MOD_GlcNHglycan	2	MOD_PKB_1	1	CLV_PCSK_PC7_1
1	TRG_NLS_MonoCore_2	2	LIG_BIR_II_1	2	LIG_LIR_Gen_1	1	MOD_N-GLC_1	2	MOD_Plk_4	1	DOC_PP2B_LxvP_1
1	TRG_NLS_MonoExtN_4	2	DOC_USP7_MATH_1	2	LIG_BIR_II_1	1	MOD_N-GLC_2	2	MOD_PRMT_GGRGG_1	1	LIG_PDZ_Class_2
		2	DOC_MAPK_NFAT4_5	2	DEG_Nend_UBRbox_2	1	MOD_ProDKin_1	2	TRG_ER_diArg_1	1	LIG_Rb_LxCxE_1
		2	DOC_MAPK_MEF2A_6	1	TRG_NLS_MonoExtC_3			2	TRG_NLS_Bipartite_1	1	LIG_TRAF2_1
		2	DEG_APCC_DBOX_1	1	MOD_SUMO_rev_2			2	TRG_NLS_MonoExtN_4	1	LIG_TRFH_1
		1	MOD_PK_1	1	MOD_SUMO_for_1			1	CLV_C14_Caspase3-7	1	MOD_CDK_SPxxK_3
		1	MOD_GlcNHglycan	1	MOD_OFUCOSY			1	CLV_PCSK_KEX2_1	1	MOD_Cter_Amidation
		1	LIG_UBA3_1	1	MOD_N-GLC_1			1	CLV_PCSK_PC1ET2_1	1	MOD_Plk_1
		1	LIG_SH2_STAT3	1	LIG_UBA3_1			1	DEG_APCC_DBOX_1	1	MOD_SUMO_rev_2
		1	LIG_PTB_Apo_2	1	LIG_TYR_ITIM			1	DOC_PP2B_LxvP_1	1	TRG_NLS_MonoExtN_4

1	LIG_PDZ_Class_1	1	LIG_TYR_ITAM	1	DOC_USP7_MATH_1
1	LIG_APCC_ABBA_1	1	LIG_TRFH_1	1	LIG_LIR_Nem_3
		1	LIG_SUMO_SIM_par_1	1	LIG_SH2_GRB2like
		1	LIG_SUMO_SIM_anti_2	1	LIG_SH3_4
		1	LIG_SH3_4	1	LIG_TRFH_1
		1	LIG_SH3_3	1	LIG_UBA3_1
		1	LIG_SH2_GRB2like	1	MOD_N-GLC_1
		1	LIG_PCNA_PIPBox_1	1	MOD_PKA_1
		1	LIG_MAD2	1	MOD_SUMO_rev_2
		1	LIG_EH_1		
		1	LIG_BRCT_BRCA1_2		
		1	DOC_USP7_MATH_1		
		1	DOC_AGCK_PIF_1		
		1	DEG_Kelch_Keap1_1		
		1	DEG_APCC_KENBOX_2		

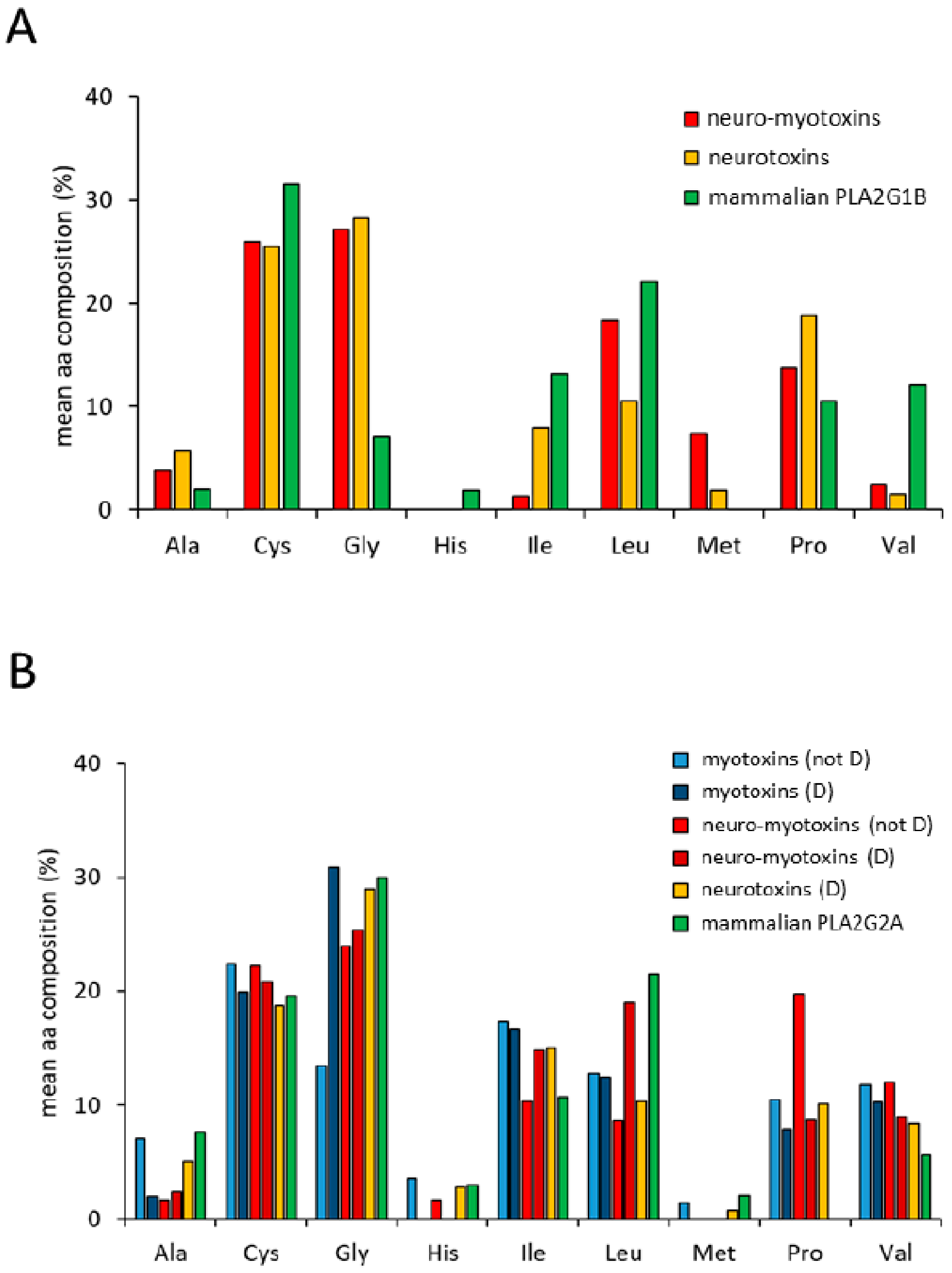

Figure S1. Percentage of presence of the amino acids A, G, V, L, I, H, P, C, M in the central region of toxins belonging to the group I and mammalian PLA2G1B homologs (panel A) and of toxins of group II and mammalian PLA2G2B homologs (panel B). Other details are described in the main text, Figure 3.

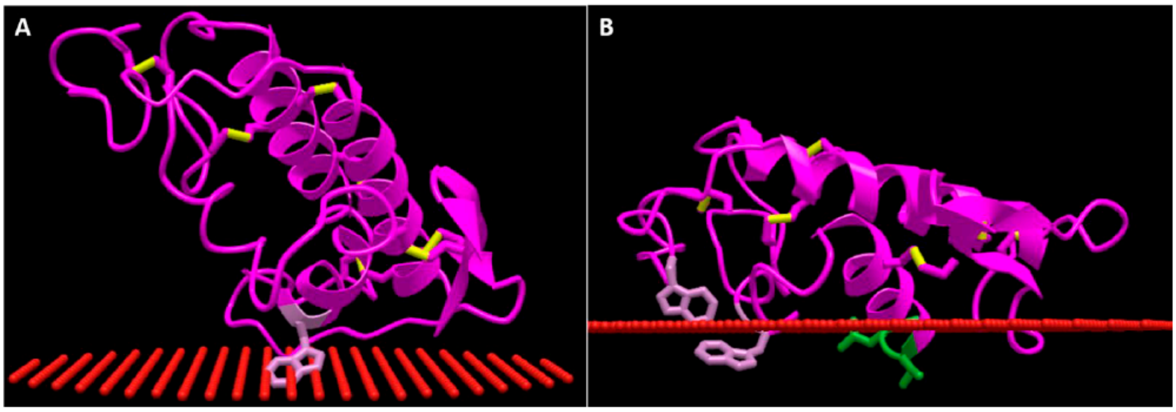

Figure S2. Inclination of PLA2G1B and notexin relative to the plasma membrane (PM). **(A)** The tryptophan in position 3 and the charged amino acids in the C-terminal region of PLA2G1B cause the protein to form an angle with the PM. (**B**) In notexin, as in other G1 toxins, tryptophan residues present in 18-19 and in the C-terminal region, together with hydrophobic amino acids in the first alpha-helix, cause the protein to assume a position parallel to the PM.
