# Supplementary material for: Short Linear Motifs Characterizing Snake Venom and Mammalian Phospholipases A2
Source: Toxins (Basel). 2021 Apr 20;13(4):290. doi: 10.3390/toxins13040290 (PMC8073766; doi:10.3390/toxins13040290)
Supplement: Supplementary file 1 [file toxins-13-00290-s001.zip › toxins-1156466 sup1.pdf]

Supplementary material

Short Linear Motifs Characterizing Snake Venom and Mammalian Phospholipases A2

Caterina Peggion and Fiorella Tonello

Clustal O sequence alignments

Group 1: 4 myotoxins, 7 neuro-myotoxins, 14 neurotoxins, 10 PLA2G1B reviewed
Gruppo 2: 61 myotoxins (14 D49), (14) neuromyotoxins (9 D49),12 neurotoxins (12 D49), 10 PLA2G2A (5 reviewed, 5 not reviewed)

Myotoxins Group 1 (all D49)

```
CLUSTAL O(1.2.4) multiple sequence alignment.

SP|P80966|PA2A1_OPHHA HLIQFGNMIQCTVPGFLSWIKYADYGCYCGAGGSGTPVDKLDRCCQVHDNCYTQAQKLPA 60
SP|C0HLF0|PA2_POROP   NLFQFRKMIKKMTK-KEPVVYYAFYGCYCGKGGRGKPKDATDRCCFVHDCCYEKVTG--- 56
SP|P81167|PA2BB_MICNI NLIDFKNMIKCTNT--RHWVSFTNYGCYCGYGGSGTPVDELDKCCQVHDKCYDTAKHVCK 58
SP|P81166|PA2BA_MICNI NLYQLKNMIKCTNT--RHWVSFTNYGCYCGYGGSGTPVDELDKCCQVHDKCYDTAKHVCK 58
                      :* :: :**:         : :: ****** ** *.* *  *:** *** **  .

SP|P80966|PA2A1_OPHHA CSSIMDSPYVKIYSYDCSERTVTCKADNDECAAFICNCDRVAAHCFAASPYNN------- 113
SP|C0HLF0|PA2_POROP   CN-----PKWGYYTYSMNKQ--IVCGGDDPCKKQVCECDKAAAICFRDNLKTYKKKYMSF 109
SP|P81167|PA2BB_MICNI CS-----PSMTMYSYDCSEGKLTCKDNNTKCKDFVCNCDRTAALCFAKAPYNN------- 106
SP|P81166|PA2BA_MICNI CS-----PSMTMYSYDCSEGKLTCKDNNTKCKDFVCNCDRTAALCFAKAPYNN------- 106
                      *.     *    *:*. .:       .:  *   :*:**:.** **     .

SP|P80966|PA2A1_OPHHA -NNYNIDTTTRC- 124
SP|C0HLF0|PA2_POROP   PNFFCTDPSEKC- 121
SP|P81167|PA2BB_MICNI -KNFKIDPTKGCQ 118
SP|P81166|PA2BA_MICNI -KNFKIDPTKGCQ 118
                       : :  * :  *
```

Neuro-Myotoxins Group 1 (all D49)

```
CLUSTAL O(1.2.4) multiple sequence alignment

SP|Q45Z47|PA22_OXYSC  NLAQFGFMIRCANGGSRSPLDYTDYGCYCGKGGRGTPVDDLDRCCQVHDECYGEAEKRLG 60
SP|P00608|PA2B_NOTSC  NLVQFSYLIQCANHGKRPTWHYMDYGCYCGAGGSGTPVDELDRCCKIHDDCYDEAGK-KG 59
SP|P00610|PA2B_HYDSC  NLVQFSYVITCANHNRRSSLDYADYGCYCGAGGSGTPVDELDRCCKIHDDCYGEAEK-QG 59
SP|P00604|PA2B3_NAJMO NLYQFKNMIHCTVP-SRPWWHFADYGCYCGRGGKGTPVDDLDRCCQVHDNCYEKAGK-MG 58
SP|Q9PSN5|PA2AE_NOTSC NLYQFGNMIQCANHGRRPTRHYMDYGCYCGKGGSGTPVDELDRCCQTHDDCYGEAEK-LP 59
SP|P00602|PA2A1_NAJMO NLYQFKNMIHCTVP-SRPWWHFADYGCYCGRGGKGTAVDDLDRCCQVHDNCYGEAEK-LG 58
SP|P00603|PA2B2_NAJMO NLYQFKNMIHCTVP-SRPWWHFADYGCYCGRGGKGTAVDDLDRCCQVHDNCYGEAEK-LG 58
                      ** **  :* *:    *   .: ******* ** ** **:*****: **:** :* *

SP|Q45Z47|PA22_OXYSC  CSP------FVTLYSWKCYGKAPSCN-TKTDCQRFVCNCDAKAAECFARSPYQKKNWNIN 113
SP|P00608|PA2B_NOTSC  CFP------KMSAYDYYCGENGPYCRNIKKKCLRFVCDCDVEAAFCFAKAPYNNANWNID 113
SP|P00610|PA2B_HYDSC  CYP------KMLMYDYYCGSNGPYCRNVKKKCNRKVCDCDVAAAECFARNAYNNANYNID 113
SP|P00604|PA2B3_NAJMO CWP------YFTLYKYKCSQGKLTCSGGNSKCGAAVCNCDLVAANCFAGARYIDANYNIN 112
SP|Q9PSN5|PA2AE_NOTSC ACNYMMSGPYYNTYSYECNEGELTCKDNNDECKAFICNCDRTAAICFARAPYNDANWNID 119
SP|P00602|PA2A1_NAJMO CWP------YLTLYKYECSQGKLTCSGGNNKCEAAVCNCDLVAANCFAGAPYIDANYNVN 112
SP|P00603|PA2B2_NAJMO CWP------YLTLYKYECSQGKLTCSGGNNKCAAAVCNCDLVAANCFAGARYIDANYNIN 112
                      .            *.: *      *   : .*   :*:**  ** ***   * . *:*::

SP|Q45Z47|PA22_OXYSC  TKARCK 119
SP|P00608|PA2B_NOTSC  TKKRCQ 119
SP|P00610|PA2B_HYDSC  TKKRCK 119
SP|P00604|PA2B3_NAJMO FKKRCQ 118
SP|Q9PSN5|PA2AE_NOTSC TKTRCQ 125
SP|P00602|PA2A1_NAJMO LKERCQ 118
SP|P00603|PA2B2_NAJMO LKERCQ 118
                       * **:
```

Neurotoxins Group 1 (all D49)

```
CLUSTAL O(1.2.4) multiple sequence alignment

SP|P00598|PA2A1_NAJAT NLYQFKNMIQCTV-PSRSWWDFADYGCYCGRGGSGTPVDDLDRCCQVHDNCYNEAEKISG 59
SP|Q9DF52|PA2B_BUNCE  NLIQFKNMIQCA--GTRPWTAYVNYGCYCGKGGSGTPVDELDRCCYTHDNCYNEAEKIPG 58
SP|Q90WA8|PA2B2_BUNFA NLLQFKNMIECA--GTRTWMAYVKYGCYCGPGGTGTPLDELDRCCQTHDQCYDNAKKFGN 58
SP|P00609|PA2B5_NOTSC NLVQFSYLIQCANHGRRPTRHYMDYGCYCGWGGSGTPVDELDRCCKIHDDCYSDAEK-KG 59
SP|P10116|PA2B2_LATCO NLIQFSELIQCANKGKRATYYYMDYGCYCGKGGSGTPVDDLDRCCKTHDDCYGQAEK-KG 59
SP|A4FS04|PA2A_NAJAT  NLYQFKNMIQCTV-PSRSWWDFADYGCYCGKGGSGTPVDDLDRCCQVHDNCYNEAEKISG 59
SP|P14556|PA2B_NAJPA  NLYQFKNMIHCTV-PSRPWWHFADYGCYCGRGGKGTPIDDLDRCCQVHDNCYEKAGK-MG 58
SP|Q90WA7|PA2B1_BUNFA NLLQFKNMIQCA--GSRLWVAYVKYGCYCGPGGTGTPLDQLDRCCQTHDHCYDNAKKFGN 58
SP|Q8UUH8|PA2BH_LATCO NLVQFSELIQCVNKGKRATYHYMDYGCYCGKGGSGTPVDALDRCCKTHDDCYGQAEK-KG 59
```

```
SP|Q8UUH9|PA2B9_LATCO NLIQFSQLIQCANKGKRPTLHYMDYGCYCGKGGSGTPVDALDRCCKTHDDCYGQAGK-KG 59
SP|Q8UUH7|PA2BK_LATCO NLIQFSQLIQCANKGKRATYHYMDYGCYCSKGGSGTPVDALDRCCKTHDDCYGQAEK-KG 59
SP|Q8UW08|PA2B0_HYDHA NLVQFSYVITCANHGRRSSLDYADYGCYCGAGGSGTPVDELDRCCQIHDDCYGEAEK-QG 59
SP|Q8UW30|PA2B7_HYDHA NLVQFSYVITCANHNRRSSLDYADYGCYCGAGGSGTPVDELDRCCKIHDDCYGEAEK-QG 59
SP|Q8UUI0|PA2B1_LATCO NLVQFSELIQCVNKGKRATYHYMDYGCYCGKGGSGTPVDALDRCCKTHDDCYGQAEK-KG 59
                      ** **. :* *.    *    : .*****. **.***:* *****  **.** .* *  .

SP|P00598|PA2A1_NAJAT CWPYFKTYSYECSQGTLTCKGGNNACAAAVCDCDRLAAICFAGAPYNNNNYNIDLKARCQ 119
SP|Q9DF52|PA2B_BUNCE  CNPNIKTYSYTCTEPNLTCTDTADTCARFLCDCDRTAAICFASAPYNSNNVMISSSTNCQ 118
SP|Q90WA8|PA2B2_BUNFA CIPYFKTYVYTCNKPDITCTGAKGSCGRTVCDCDRAAALCFAAAPYNLANFGINKETHCQ 118
SP|P00609|PA2B5_NOTSC CSPKMSAYDYYCGENGPYCRNIKKKCLRFVCDCDVEAAFCFAKAPYNNANWNIDTKKRCQ 119
SP|P10116|PA2B2_LATCO CFPFLTLYNFICFPGGPTCDR-GTTCQRFVCDCDIQAAFCFARSPYNNKNYNINISKRCK 118
SP|A4FS04|PA2A_NAJAT  CWPYFKTYSYECSQGTLTCKGGNNACAAAVCDCDRLAAICFAGAPYTDANYNIDLKARCQ 119
SP|P14556|PA2B_NAJPA  CWPYFTLYKYKCSKGTLTCNGRNGKCAAAVCNCDLVAANCFAGAPYINANYNIDFKKRCQ 118
SP|Q90WA7|PA2B1_BUNFA CIPYFKTYEYTCNKPDLTCTDAKGSCARNVCDCDRAAAICFAAAPYNLANFGINKETHCQ 118
SP|Q8UUH8|PA2BH_LATCO CFPFLTLYNFGCFPGGPTCGK-GNTCQRFVCDCDLKAALCFAKSPYNNNNYNIDTKKRCK 118
SP|Q8UUH9|PA2B9_LATCO CIPFVTLYNFGCFPGAPQCGK-GNTCQRFVCACDLKAALCFAKSPYNNNNYNIDTKKKCQ 118
SP|Q8UUH7|PA2BK_LATCO CFPLLSLYNFACFPGAPQCGK-GNTCQRFVCACDLKAALCFAKSPYNN-NYNIDIKKKCQ 117
SP|Q8UW08|PA2B0_HYDHA CYPKMLIYDYYCGSDGPYCRNVKKKCNRMVCDCDVAAAKCFARNAYNNANYNIDTNKRCK 119
SP|Q8UW30|PA2B7_HYDHA CYPKMLIYDYDCGSNGPYCKNVTKKCNRKVCDCDVAAAKCFARNAYNNANYNIDTKKRCK 119
SP|Q8UUI0|PA2B1_LATCO CFPLLSLYNFACFPGAPQCGK-GNTCQRFVCACDLKAALCFAKSPYNNNNYNIDIKKKCQ 118
                      * * .  * : *      *      *   :* **  ** ***   *   *  *. . .*:

SP|P00598|PA2A1_NAJAT ----------
SP|Q9DF52|PA2B_BUNCE  ----------
SP|Q90WA8|PA2B2_BUNFA ----------
SP|P00609|PA2B5_NOTSC ----------
SP|P10116|PA2B2_LATCO ----------
SP|A4FS04|PA2A_NAJAT  ----------
SP|P14556|PA2B_NAJPA  ----------
SP|Q90WA7|PA2B1_BUNFA ----------
SP|Q8UUH8|PA2BH_LATCO ----------
SP|Q8UUH9|PA2B9_LATCO TLIYARLQTQ 128
SP|Q8UUH7|PA2BK_LATCO TLIYMRLQTQ 127
SP|Q8UW08|PA2B0_HYDHA ----------
SP|Q8UW30|PA2B7_HYDHA ----------
SP|Q8UUI0|PA2B1_LATCO TLIYMRLQTQ 128
```

PLA2G1B reviewed (PLA2G1B of camel has been removed because it is very different).

```
CLUSTAL O(1.2.4) multiple sequence alignment

SP|P04054|PA21B_HUMAN AVWQFRKMIKCVIPGSDPFLEYNNYGCYCGLGGSGTPVDELDKCCQTHDNCYDQAKKLDS 60
SP|Q9Z0Y2|PA21B_MOUSE AVWQFRNMIKCTIPGSDPLKDYNNYGCYCGLGGWGTPVDDLDRCCQTHDHCYSQAKKLES 60
SP|P06596|PA21B_CANLF AVWQFRNMIKCTIPESDPLKDYNDYGCYCGLGGSGTPVDELDKCCQTHDHCYSEAKKLDS 60
SP|P04055|PA21B_RAT   AVWQFRNMIKCTIPGSDPLREYNNYGCYCGLGGSGTPVDDLDRCCQTHDHCYNQAKKLES 60
SP|P00594|PA21B_HORSE AVWQFRSMIQCTIPNSKPYLEFNDYGCYCGLGGSGTPVDELDACCQVHDNCYTQAKELSS 60
SP|P00593|PA21B_BOVIN ALWQFNGMIKCKIPSSEPLLDFNNYGCYCGLGGSGTPVDDLDRCCQTHDNCYKQAKKLDS 60
SP|P43434|PA21B_CAVPO ALWQFRDMIKCAIPGSRPYSEYNNYGCFCGLGGSGTPVDELDRCCEIHDACYTQAKHLES 60
SP|P00592|PA21B_PIG   ALWQFRSMIKCAIPGSHPLMDFNNYGCYCGLGGSGTPVDELDRCCETHDNCYRDAKNLDS 60
SP|Q7M334|PA21B_RABIT ALWQFRGMIQCTIPGSSPYLEFNGYGCYCGLGGSGTPVDELDRCCQTHDQCYTQAKKLSS 60
SP|P14419|PA21B_SHEEP ALWQFNGMIKCKIPSSEPLLDFNNYGCYCGLGGSGTPVDDLDRCCQTHDNCYKQAKKLDS 60
                      *:***. **:* ** * *  ::*.***:***** *****:** **: ** ** :**.*.*

SP|P04054|PA21B_HUMAN CKFLLDNPYTHTYSYSCSGSAITCSSKNKECEAFICNCDRNAAICFSKAPYNKAHKNLDT 120
SP|Q9Z0Y2|PA21B_MOUSE CKFLIDNPYTNTYSYSCSGSEITCSAKNNKCEDFICNCDREAAICFSKVPYNKEYKNLDT 120
SP|P06596|PA21B_CANLF CKFLLDNPYTKIYSYSCSGSEITCSSKNKDCQAFICNCDRSAAICFSKAPYNKEHKNLDT 120
SP|P04055|PA21B_RAT   CKFLIDNPYTNTYSYKCSGNVITCSDKNNDCESFICNCDRQAAICFSKVPYNKEYKDLDT 120
SP|P00594|PA21B_HORSE CRFLVDNPYTESYKFSCSGTEVTCSDKNNACEAFICNCDRNAAICFSKAPYNPENKNLDS 120
SP|P00593|PA21B_BOVIN CKVLVDNPYTNNYSYSCSNNEITCSSENNACEAFICNCDRNAAICFSKVPYNKEHKNLDK 120
SP|P43434|PA21B_CAVPO CKSVIDNPYTNSYSFSCSGTNIICSSKNKECEEFICNCDRAAAICFSKAPYNENNKNINK 120
SP|P00592|PA21B_PIG   CKFLVDNPYTESYSYSCSNTEITCNSKNNACEAFICNCDRNAAICFSKAPYNKEHKNLDT 120
SP|Q7M334|PA21B_RABIT CSFLVDNPYTNSYSYSCSGTTVTCSSKNKECEAFICDCDRKAAICFSKRPYNKEYKPISK 120
SP|P14419|PA21B_SHEEP CKVLVDNPYTNSYSYSCSNKQITCSSENNACEAFICNCDRNAAICFSEVPYNNEHKNLDK 120
                      *  ::*****. *.:.**.. : *. :*: *: ***:*** ******: ***   * :..

SP|P04054|PA21B_HUMAN KKYCQS 126
SP|Q9Z0Y2|PA21B_MOUSE GKFC-- 124
SP|P06596|PA21B_CANLF KKYC-- 124
SP|P04055|PA21B_RAT   KKHC-- 124
SP|P00594|PA21B_HORSE KRKCA- 125
SP|P00593|PA21B_BOVIN KNC--- 123
SP|P43434|PA21B_CAVPO KERC-- 124
SP|P00592|PA21B_PIG   KKYC-- 124
SP|Q7M334|PA21B_RABIT --YC-- 122
SP|P14419|PA21B_SHEEP KYC--- 123
```

Myotoxins Group 2, NOT family:"D49 sub-subfamily"

```
NOT D49 CLUSTAL O(1.2.4) multiple sequence alignment
```

```
SP|P24605|PA2H2_BOTAS SLFELGKMILQETGKNPAKSYGAYGCNCGVLGRGKPKDATDRCCYVHKCCYKKLTGCNPK 60
SP|P0DJJ9|PA2B2_PROFL SLVQLWKMIFQETGKEAAKNYGLYGCNCGVGRRGKPKDATDSCCYVHKCCYKKVTGCNPK 60
SP|Q90249|PA2B1_BOTJR SLFELGKMILQETGKNPAKSYGAYGCNCGVLGRGKPKDATDRCCYVHKCCYKKLTGCDPK 60
SP|P82114|PA2H1_BOTMO SLVELGKMILQETGKNPAKSYGAYGCNCGVLGRGKPKDATDRCCYVHKCCYKKLTNCDPK 60
SP|P80963|PA2H_BOTSC  SMYELGKMILLETGKNAATSYIAYGCNCGVGRRGQPLDATDRCCYVHKCCYKKLTGCNPL 60
SP|I6L8L6|MTX2_BOTBZ  SLFQLGKMILQETGKNPAASYGAYGCNCGVLGRGKPKDATDRCCYVHKCCKKKLTGCDPK 60
SP|O57385|PA2H_DEIAC  SLFELGKMIWQETGKNPVKNYGLYGCNCGVGGRGEPLDATDRCCFVHKCCYKKLTDCDSK 60
SP|P49121|PA2H1_AGKCL SLLELGKMILQETGKNAITSYGSYGCNCGWGHRGQPKDATDRCCFVHKCCYKKLTDCNHK 60
SP|P04361|PA2HB_AGKPI SVLELGKMILQETGKNAITSYGSYGCNCGWGHRGQPKDATDRCCFVHKCCYKKLTDCNHK 60
SP|Q6JK69|PA2H1_BOTAT SLVELGKMILQETGKNPLTSYGAYGCNCGVGGRGKPKDATDRCCYVHKCCYKKMTDCDPK 60
SP|Q9I834|PA2H2_BOTMO SLFELGKMILQETGKNPAKSYGVYGCNCGVGGRGKPKDATDRCCYVHKCCYKKLTGCDPK 60
SP|P84776|PA2H_PROMB  SLIELTKMVFQETGKNPVTYYTLYGCNCGVGRRGKPKDATDRCCFVHRCCYKKLTGCDPK 60
SP|Q2PWA3|PA2HP_PROMU SVIELGKMVFQETGKNPVKNYGLYGCNCGVGKRGKPVDATDSCCFVHRCCYKKVTGCDPK 60
SP|P0DJJ8|PA2B1_PROFL SLVQLWKMIFQETGKEAAKNYGLYGCNCGVGRRGKPKDATDSCCYVHKCCYKKVTGCDPK 60
SP|Q2YHJ9|PA2HA_TRIPE SVIQLGKMILQETGKNPVKYYGAYGCNCGPLGRRKPLDATDRCCYMHKCCYKKLTDSNPI 60
SP|Q2YHJ8|PA2HB_TRIPE SVIQLGKMILQETGKNPVKYYGAYGCNCGPLGRRKPLDATDRCCYMHKCCYKKLTDSNPI 60
SP|Q2YHJ4|PA2HB_TRIBO SVIELGKMILQETGKNPVTYYSAYGCNCGPLGRRKPLDATDRCCFMHKCCYKKLTDSNPI 60
SP|B5U6Y4|PA2HS_ECHOC SVVELGKMIIQETGKSPFPSYTSYGCFCGGGEKGTPKDATDRCCFVHSCCYDKLPDCSPK 60
SP|C0HKC1|PA2HB_AGKPC SVLELGKMILQETGKNAITSYGSYGCNCGWGHRGQPKDATDRCCFVHKCCYKKLTDCNHK 60
SP|Q6H3D4|PA2HE_TRIST SVIELGKMIFQETGKNPATSYGLYGCNCGPGGRRKPKDATDRCCYVHKCCYKKLTDCDPI 60
SP|Q6H3D5|PA2HI_TRIST SLVQLGKMIFQETGKNPATSYGLYGCNCGPGGRRKPKDATDRCCFLHKCCYKKLTDCDPI 60
SP|C0HKC2|PA2HB_AGKPL SVLELGKMILQETGKNAITSYGSYGCNCGWGHRGQPKDATDRCCFVHKCCYKKLTDCNHK 60
SP|C7G1G6|PA2B3_PROFL SLVQLWKMIFQETGKEAAKNYGLYGCNCGVGRRGKPKDATDSCCYVHKCCYKKVTGCNPK 60
SP|Q6H3D6|PA2HD_TRIST HLLQLRKMIKKMTNKEPILSYGKYGCNCGMAGRGQPVDGTDRCCSIHNCCYGKVNGCSPK 60
SP|P0DTS8|BBTX2_BOTBZ SLFELGKMILQETGKNPAKSYGAYGCYCGVLGRGKPKDATDRCCYVHKCCYKKLTGCDNK 60
                        : :* **:    *.*.     *   *** **    :  * *.** ** :* **   *: ...

SP|P24605|PA2H2_BOTAS KDRYSYSWKDKTIVCGENN-SCLKELCECDKAVAICLRENLNTYNKKYRYY-LKPLCKKA 118
SP|P0DJJ9|PA2B2_PROFL MDSYSYSWKNKAIVCGEKNPPCLKQVCECDKAVAICLRENLGTYNKKYT-IYPKPFCKKA 119
SP|Q90249|PA2B1_BOTJR KDRYSYSWKDKTIVCGENN-PCLKELCECDKAVAICLRENLGTYNKKYRYH-LKPFCKKA 118
SP|P82114|PA2H1_BOTMO KDRYSYDWKNKTIVCGEEN-PCLKQLCECDKAVAICLRENKGTYNKK-RDVYLKPFCDKG 118
SP|P80963|PA2H_BOTSC  TDRYSHSLKNKTIVCGENK-PCLKEMCECDKALAICLGKNVNTYNKNYKI-TMKMFCKKP 118
SP|I6L8L6|MTX2_BOTBZ  KDRYSYSWKDKTIVCGENN-PCLKELCECDKAVAICLRENLNTYNKKYRYH-LKPFCKKA 118
SP|O57385|PA2H_DEIAC  KDRYSYKWKNKAIVCGKNQ-PCMQEMCECDKAFAICLRENLDTYNKSFRYH-LKPSCKKT 118
SP|P49121|PA2H1_AGKCL TDRYSYSWKNKAIICEEKN-PCLKEMCECDKAVAICLRENLDTYNKKYKA-YFKFKCKKP 118
SP|P04361|PA2HB_AGKPI TDRYSYSWKNKAIICEEKN-PCLKEMCECDKAVAICLRENLDTYNKKYKA-YFKLKCKKP 118
SP|Q6JK69|PA2H1_BOTAT KDRYSYSWKDKTIVCGEKN-SCLKELCECDKAVAICLRENLDTYNKKYKNNYLKPFCKKA 119
SP|Q9I834|PA2H2_BOTMO KDRYSYSWKDKTIVCGENN-SCLKELCECDKAVAICLRENLDTYNKKYRYNYLKPFCKKA 119
SP|P84776|PA2H_PROMB  KDRYSYSWENKAIVCGEKN-PCLKELCECDKAVAICLRKNLGTYDKNYRF-TMKFLCDKP 118
SP|Q2PWA3|PA2HP_PROMU KDRYSYSWENKAIVCGEKNPPCLKQVCECDKAVAICLRENLGTYDKKHRV-TMKFLCKAP 119
SP|P0DJJ8|PA2B1_PROFL MDSYSYSWKNKAIVCGEKNPPCLKQVCECDKAVAICLRENLGTYNKKYT-IYPKPFCKKA 119
SP|Q2YHJ9|PA2HA_TRIPE KDRYSYSWENKAIVCKEKN-PRLKEMCECDKAVAICFRENMRTYNKKERI-NTKIFCKKT 118
SP|Q2YHJ8|PA2HB_TRIPE KDSYSYSWENKAIVCKEKN-PRLKEMCECDKAVAICFRENMDTYNKKERI-NTKIFCKKT 118
SP|Q2YHJ4|PA2HB_TRIBO KDSYSYSWENKAIVCKEKN-PRLKEMCECDKAVAICFRENMGTYNKKERI-NTKIFCKKT 118
SP|B5U6Y4|PA2HS_ECHOC TDRYKYQRENGEIICENST-SCKKRICECDKAVAVCLRENLQTYNKKYTY-YPNFLCKGE 118
SP|C0HKC1|PA2HB_AGKPC TDRYSYSWKNKAIICEEKN-PCLKEMCECDKAVAICLRENLDTYNKKYKA-YFKLKCKKP 118
SP|Q6H3D4|PA2HE_TRIST KDRYSYSWVNKAIVCGEDN-PCLKEMCECDKAVAICFRENLDTYDKKKKI-NLKLFCKKT 118
SP|Q6H3D5|PA2HI_TRIST KDSYSYSWVNKAIVCGGDD-PHLKEMCECDKAMAICFRENLDTYDKKKKI-NLKLFCKKT 118
SP|C0HKC2|PA2HB_AGKPL TDRYSYSWKNKAIICEEKN-PCLKEMCECDKAVAICLRENLDTYNKKYKA-YFKLKCKKP 118
SP|C7G1G6|PA2B3_PROFL MDSYSYSWKNKAIVCGENNPPCLKQVCECDKAVAICLRENLGTYNKKYT-IYPKPFCKKA 119
SP|Q6H3D6|PA2HD_TRIST WDYYTYSEENGDIVCEEKH-PC-KDVCECDKAVATCFRDNLDTYKKRNIF-HPKSSCVKV 117
SP|P0DTS8|BBTX2_BOTBZ KDRYSYSWKDKTIVCGENN-PCLKELCECDKAVAICLRENLNTYNKKYRYH-LKPLCKKA 118
                       * *.:.  :  *:*  .      : :******.* *: .*   **.*        :  *

SP|P24605|PA2H2_BOTAS D-AC 121
SP|P0DJJ9|PA2B2_PROFL DTC- 122
SP|Q90249|PA2B1_BOTJR D-PC 121
SP|P82114|PA2H1_BOTMO R-DC 121
SP|P80963|PA2H_BOTSC  DAC- 121
SP|I6L8L6|MTX2_BOTBZ  D-PC 121
SP|O57385|PA2H_DEIAC  SEQC 122
SP|P49121|PA2H1_AGKCL ETC- 121
SP|P04361|PA2HB_AGKPI DTC- 121
SP|Q6JK69|PA2H1_BOTAT D-AC 122
SP|Q9I834|PA2H2_BOTMO D-PC 122
SP|P84776|PA2H_PROMB  EKC- 121
SP|Q2PWA3|PA2HP_PROMU ESC- 122
SP|P0DJJ8|PA2B1_PROFL DTC- 122
SP|Q2YHJ9|PA2HA_TRIPE PEPC 122
SP|Q2YHJ8|PA2HB_TRIPE PEPC 122
SP|Q2YHJ4|PA2HB_TRIBO SEPC 122
SP|B5U6Y4|PA2HS_ECHOC PEKC 122
SP|C0HKC1|PA2HB_AGKPC DTC- 121
SP|Q6H3D4|PA2HE_TRIST SEQC 122
SP|Q6H3D5|PA2HI_TRIST SEQC 122
SP|C0HKC2|PA2HB_AGKPL DTC- 121
SP|C7G1G6|PA2B3_PROFL DTC- 122
SP|Q6H3D6|PA2HD_TRIST STPC 121
SP|P0DTS8|BBTX2_BOTBZ D-AC 121
```

Myotoxins Group 2, family:"D49 sub-subfamily"

```
D49 CLUSTAL O(1.2.4) multiple sequence alignment

SP|P0DTS7|PA23_BOTBZ  SLWEWGQMILKETGKNPFPYYGAYGCYCGWGGRRKPKDATDRCCFVHDCCRYKKLTGCPK 60
SP|P20474|PA2B3_BOTAS SLIEFAKMILEETKRLPFPYYTTYGCYCGWGGQGQPKDATDRCCFVHDCC-YGKLSNCKP 59
SP|Q71QE8|PA2BN_CROVV NLLQFNKMIKMMTKKNAFPFYTSYGCYCGWGGRGRPKDATDRCCFVHDCC-YEKLTNCSP 59
SP|Q7ZTA7|PA2AD_CROOA SLVQFEMLIMKVAKRSGLFSYSAYGCYCGWGGHGRPQDATDHCCFVHDCC-YGKVTDCNP 59
SP|P86806|PA2BA_CRODM SLLQFNKMIKFETRKSGVPFYAAYGCYCGWGGR-RPKDPTDRCCFVHDCC-YGKLTKCNT 58
SP|P06859|PA2A1_PROFL GLWQFENMIIKVVKKSGILSYSAYGCYCGWGGRGKPKDATDRCCFVHDCC-YGKVTGCNP 59
SP|P45881|PA2B2_BOTJR DLWQFGQMILKETGKLPFPYYTTYGCYCGWGGQGQPKDATDRCCFVHDCC-YGKLTNCKP 59
SP|P86805|PA2B9_CRODM SLVQFNKMIKFETRKSGLPFYAAYGCYCGWGGQ-RPKDATDRCCFVHDCC-YGKVAKCNT 58
SP|Q6EER4|PA2B_BOTSC  NLLQFNKMIKIMTRKNGIPYYSSYGCYCGWGGQGQPLDATDRCCFVHDCC-YEKLTDCSP 59
SP|Q9PVF4|PA2BD_CALRH SMFNLWKMIMVMTGKEATKNYGMYGCNCGPMKRGKPKDATDQCCADHDCC-YKKLTDCDP 59
SP|Q8QG87|PA2A_BOTIN  NLWQFGKMMNYVMGQSVVYKYFYYGCYCGWGGIGQPRDATDRCCFVHDCC-YGKVTGCDP 59
SP|Q6EER5|PA2B_CERGO  NLLQFNKMIKIMTKKNAVPFYTSYGCYCGWGGRGKPKDATDRCCFEHDCC-YEKLTDCSP 59
SP|P58464|PA2B3_BOTPI DLWQFGQMILKETGKLPFPYYTYGGCYCGVGGRRGLGTKDDRCCYVHDCC-YKKLTGCPK 59
SP|Q6EER6|PA2B_SISMS  NLLQFNKMIKIMTKKNAIPSYTSYGCYCGWGGRGRPKDATDRCCFVHDCC-YEKLTDCSP 59
                      .: :   ::     :     *   ** **           *:**  **** * *:: *

SP|P0DTS7|PA23_BOTBZ  TNDRYSYSRLDYTIVCGEDDPCK-EICECDKAAAVCFRENLRTYN-KKYMAHLRVLCKKD 118
SP|P20474|PA2B3_BOTAS KTDRYSYSRKSGVIICGEGTPCEKQICECDKAAAVCFRENLRTYK-KRYMAYPDLLCKKP 118
SP|Q71QE8|PA2BN_CROVV KTDIYSYSWKRGVIICGKGTPCEKQICECDRAAAVCFRENLPTYK-KRYMFYLDFLCTDP 118
SP|Q7ZTA7|PA2AD_CROOA KTASYTYSEENGEIVCGGDDPCKKQVCECDRVAAICFRDNIPTYD-NKYWRFPPENCQEE 118
SP|P86806|PA2BA_CRODM KWDIYSYSLKSGYITCGKGTWCKEQICECDRVAAECLRRSLNTYK-NEYMFYPDSRCRGP 117
SP|P06859|PA2A1_PROFL KLGKYTYSWNNGDIVCEGDGPCK-EVCECDRAAAICFRDNLDTYDRNKYWRYPASNCQED 118
SP|P45881|PA2B2_BOTJR KTDRYSYSRENGVIICGEGTPCEKQICECDKAAAVCFRENLRTYK-KRYMAYPDVLCKKP 118
SP|P86805|PA2B9_CRODM KWDIYSYSLKSGYITCGKGTWCKEQICECDRVAAECLRRSLSTYK-NEYMFYPDSRCREP 117
SP|Q6EER4|PA2B_BOTSC  KTDIYSYSWKSGVIICGEGTPCEKQICECDRVAAVCFGANLGTYK-KSYMFYPDFLCTEP 118
SP|Q9PVF4|PA2BD_CALRH KKESYSYKFEKGEILCGETNPCLNQACECDKAVATCFRDNLDTYN-KKQQFNTGIFCSKA 118
SP|Q8QG87|PA2A_BOTIN  KTDSYTYSKENGDVVCGGDDPCKKQICECDRVAATCFRDNKDTYD-MKYWLYGAKNCQEE 118
SP|Q6EER5|PA2B_CERGO  KTDIYSYSWKSGVIICGEGTPCEKQICECDRVAAVCFGTNLRTYK-KRYMFYPDFLCTDP 118
SP|P58464|PA2B3_BOTPI TDDRYSYSWLDLTIVCGEDDPCK-ELCECDKAIAVCFRENLGTYN-KKYRYHLKPCKKAD 117
SP|Q6EER6|PA2B_SISMS  KTDIYSYSWKSGVITCGEGTPCEKQICECDRAAAVCFGENLPTYK-KRYMFYPDFLCTDP 118
                      .   *:*.     : *     *  : ****:. * *:  .  **.

SP|P0DTS7|PA23_BOTBZ  KPC-- 121
SP|P20474|PA2B3_BOTAS AEKC- 122
SP|Q71QE8|PA2BN_CROVV SEKC- 122
SP|Q7ZTA7|PA2AD_CROOA PEPC- 122
SP|P86806|PA2BA_CRODM PEYTC 122
SP|P06859|PA2A1_PROFL SEPC- 122
SP|P45881|PA2B2_BOTJR AEKC- 122
SP|P86805|PA2B9_CRODM PEYTC 122
SP|Q6EER4|PA2B_BOTSC  SEKC- 122
SP|Q9PVF4|PA2BD_CALRH KAC-- 121
SP|Q8QG87|PA2A_BOTIN  SEPC- 122
SP|Q6EER5|PA2B_CERGO  SEKC- 122
SP|P58464|PA2B3_BOTPI KPC-- 120
SP|Q6EER6|PA2B_SISMS  SEKC- 122
```

Neuro-Myotoxins Group II, NOT family:"D49 sub-subfamily" (5)

```
NOT D49 CLUSTAL O(1.2.4) multiple sequence alignment

SP|Q9IAT9|PA2H_BOTPA  SLFELGKMILQETGKNPAKSYGAYGCNCGVLGRGQPKDATDRCCYVHKCCYKKLTGCDPK 60
SP|P58399|PA2H1_BOTPI SLFELGKMILQETGKNPAKSYGAYGCNCGVLGRGKPKDATDRCCYVHKCCYKKLTGCNPK 60
SP|P17935|PA2HL_VIPAA SVIEFGKMIQEETDKNPLTSYSFYGCHCGLGNKGKPKDATDRCCFVHSCCYAKLPDCSPK 60
SP|P86453|PA2HB_BOTAL SLFELGKMILQETGKNPAKSYGAYYCYCGWGGQGQPKDATDRCCYVHKCCYKKLTGCNPK 60
SP|P0DKU1|PA2H_GLOUS  SLLQFRKMIKKMTGKEPVVSYAFYGCYCGSGGRGKPKDATDRCCFVHQCCYEKVTGCDPK 60
                      *:::: *** : *.*:*  **. * * **  .:*:*********:**.*** *: .*.**

SP|Q9IAT9|PA2H_BOTPA  KDRYSYSWKDKTIVCGENNPCLKELCECDKAVAICLRENLGTYNKKYRYHLKPFCKKADP 120
SP|P58399|PA2H1_BOTPI KDRYSYSWKDKTIVCGENNPCLKELCECDKAVAICLRENLGTYNKLYRYHLKPFCKKADD 120
SP|P17935|PA2HL_VIPAA TNRYEYHRENGAIVCGSSTPCKKQICECDRAAAICFRENLKTYNKKYKVYLRFKCKGVSE 120
SP|P86453|PA2HB_BOTAL KDRYSYSWKDKTIVCGENNSCLKELCECDKAVAICLRENLNTYNKKYRYYLKPLCKKADA 120
SP|P0DKU1|PA2H_GLOUS  WDDYTYSWKDGDIVCGGDDPCKKEVCECDRAAAICFRDNLKTYKKIYMAYPDIFCSSKSE 120
                       : * *  ::  ****  .  * *::****:*.***:*:** **:* *  :    *.  .

SP|Q9IAT9|PA2H_BOTPA  C- 121
SP|P58399|PA2H1_BOTPI C- 121
SP|P17935|PA2HL_VIPAA KC 122
SP|P86453|PA2HB_BOTAL C- 121
SP|P0DKU1|PA2H_GLOUS  KC 122
```

Neuro-Myotoxins Group II, family:"D49 sub-subfamily" (11, two discarded as incomplete)

```
D49 CLUSTAL O(1.2.4) multiple sequence alignment

SP|Q90W39|PA2BT_PROMU NLLQFNKMIKIMTKKNAIPFYSSYGCYCGWGGQG-KPKDATDRCCFVHDCCYGKLTDCSP 59
SP|P59071|PA2B8_DABRR SLLEFGKMILEETGKLAIPSYSSYGCYCGWGGKG-TPKDATDRCCFVHDCCYGNLPDCNP 59
SP|P86804|PA2B2_BOTMA DLWQWGQMILKETGKIPFSYYGAYGCYCGWGGRGGKPKAGTDRCCYVHDCCYGKLTSCPK 60
SP|P86803|PA2B1_BOTMA DLWQFGQMILKETGKIPFPYYGAYGCYCGWGGRGGKPKAGTDRCCYVHDCCYGKLTSCPK 60
```

```
SP|Q805A2|PA2BN_PROFL NLLQFNKMIKIMTKKNGFPFYTSYGCYCGWGGRG-KPKDATDRCCFVHDCCYEKLTDCSP 59
SP|P0C8M1|PA2B1_BOTMO DLWQFNKMIKKEVGKLPFPFYGAYGCYCGWGGRGEKPKDGTDRCCFVHDCCYKKLTGCPK 60
SP|P0CAS4|PA2BD_CRODR SLVQFEKMIKEETGKNAVPFYAFYGCYCGWGGRG-RPKDATDRCCIVHDCCYEKLVKCNT 59
SP|P0CAS3|PA2BC_CRODR SLLQFNKMIKFETRKNAIPFYAFYGCYCGWGGQG-RPKDATDRCCIVHDCCYGKLAKCNT 59
SP|Q6EER3|PA2B2_SISTE NLLQFNKMIKIMTKKNAIPSYSSYGCYCGWGGRG-RPKDATDRCCFVHDCCYEKLTDCSP 59
                      .* :: :**   . *  .  *  *********:*  ** .***** ****** :*  *

SP|Q90W39|PA2BT_PROMU KSDIYSYSWKTGIIICGEGTECEKKICECDRAAAVCLGHNLRTYKKRYMFYPDFLCTDPS 119
SP|P59071|PA2B8_DABRR KSDRYKYKRVNGAIVCEKGTSCENRICECDKAAAICFRQNLNTYSKKYMLYPDFLCKGEL 119
SP|P86804|PA2B2_BOTMA TDDRYSYSRLDLTIVCGEDDPCK-ELCECDKKIAVCFRENLGTYNKKYRYHLKS-CKKAD 118
SP|P86803|PA2B1_BOTMA TDDRYSYSWLDGTIVCGEDDPCK-ELCECDKKIAVCFRENLGTYNKKYRYHLKS-CKKAD 118
SP|Q805A2|PA2BN_PROFL KSDIYSYSWKTGVIICGEGTECEKQICECDRAAAVCFGQNLRTYKKKYMFYPDFLCTDPT 119
SP|P0C8M1|PA2B1_BOTMO WDDRYSYSWKDITIVCGEDLPCE-EICECDRAAAVCFYENLGTYNKKYMKHLKP-CKKAD 118
SP|P0CAS4|PA2BD_CRODR KWDFYRYSLRSGYFQCGKGTWCEQQICECDRVAAECLRRSLSTYRYGKMIYPDSRCREPS 119
SP|P0CAS3|PA2BC_CRODR KWDFYRYSLRSGYFQCGKGTWCEQQICECDRVAAECLRRSLSTYRYGYMIYPDSRCREPS 119
SP|Q6EER3|PA2B2_SISTE KTDTYSYSLKSGVIICGGNDPCKKQICECDKAAAVCFGENLSTYKKRYMFYPDFLCTDPS 119
                        * * *.     : *  .  *: .:****:  * *: ..* **      : .  *

SP|Q90W39|PA2BT_PROMU EKC 122
SP|P59071|PA2B8_DABRR KC- 121
SP|P86804|PA2B2_BOTMA KPC 121
SP|P86803|PA2B1_BOTMA KPC 121
SP|Q805A2|PA2BN_PROFL EKC 122
SP|P0C8M1|PA2B1_BOTMO YPC 121
SP|P0CAS4|PA2BD_CRODR ETC 122
SP|P0CAS3|PA2BC_CRODR ETC 122
SP|Q6EER3|PA2B2_SISTE ETC 122
```

Neurotoxins Group II (all D49)

```
CLUSTAL O(1.2.4) multiple sequence alignment

SP|P00626|PA2BA_VIPAA SLLEFGMMILGETGKNPLTSYSFYGCYCGVGGKGTPKDATDRCCFVHDCCYGNLPDCSPK 60
SP|P86169|PA2BA_CRODR HLLQFNKMIKFETRKNAIPFYAFYGCYCGWGGRGRPKDATDRCCFVHDCCYGKLAKCNTK 60
SP|P11407|PA2BC_VIPAA SLLEFGMMILGETGKNPLTSYSFYGCYCGVGGKGTPKDATDRCCFVHDCCYGNLPDCSPK 60
SP|P14424|PA2BB_VIPAA SLLEFGMMILGETGKNPLTSYSFYGCYCGVGGKGTPKDATDRCCFVHDCCYGNLPDCSPK 60
SP|P0CAS5|PA2BE_CRODU HLLQFNKMIKFETRKNAVPFYAFYGCYCGWGGQRRPKDATDRCCFVHDCCYGKLTKCNTK 60
SP|P14421|PA2N_GLOHA  NLLQFNKMIKEETGKNAIPFYAFYGCYCGGGGQGKPKDGTDRCCFVHDCCYGRLVNCNTK 60
SP|P00622|PA2B_BITCA  NLIQFGNMISAMTGKSSL-AYASYGCYCGWGGKGQPKDDTDRCCFVHDCCYGKADKCSPK 59
SP|P0CAS6|PA2BF_CRODU SLLQFNKMIKFETRKNAVPFYAFYGCYCGWGGRRRPKDATDRCCFVHDCCYEKVTKCNTK 60
SP|Q1ZY03|PA2B_DEIAC  HLLQFNKMIKIMTRKNAFPFYTSYGCYCGWGGRGWPKDATDSCCFVHDCCYQKLTGCSPK 60
SP|P04417|PA2B_GLOBL  HLLQFRKMIKKMTGKEPVISYAFYGCYCGSGGRGKPKDATDRCCFVHDCCYEKVTGCKPK 60
SP|A8CG84|PA2BS_DABSI SLLEFGKMILEETGKLAIPSYSSYGCYCGWGGKGTPKDATDRCCFVHDCCYGNLPDCNPK 60
SP|A8CG87|PA2A2_DABRR NLYQFGEMINQKTGNFGLLSYVYYGCYCGWGGKGKPQDATDRCCFVHDCCYGRVKGCDPK 60
                       * :*  **   * :  .  *  ****** **:  *:* ** ********* .   *. *

SP|P00626|PA2BA_VIPAA TDRYKYHRENGAIVCGKGTSCENRICECDRAAAICFRKNLKTYNYIYRNYPDFLCKKESE 120
SP|P86169|PA2BA_CRODR WDIYPYSLKSGYITCGKGTWCEEQICECDRVAAECLRRSLSTYKYGYMFYPDSRCRGPSE 120
SP|P11407|PA2BC_VIPAA TDRYKYHRENGAIVCGKGTSCENRICECDRAAAICFRKNLKTYNYIYRNYPDILCKEESE 120
SP|P14424|PA2BB_VIPAA TDRYKYHRENGAIVCGKGTSCENRICECDRAAAICFRKNLKTYNHIYMYYPDFLCKKESE 120
SP|P0CAS5|PA2BE_CRODU WDIYRYSLKSGYITCGKGTWCKEQICECDRVAAECLRRSLSTYKNEYMFYPKSRCRRPSE 120
SP|P14421|PA2N_GLOHA  SDIYSYSLKEGYITCGKGTNCEEQICECDRVAAECFRRNLDTYNNGYMFYRDSKCTETSE 120
SP|P00622|PA2B_BITCA  MILYSYKFHNGNIVCGDKNACKKKVCECDRVAAICFAASKHSYNKNLWRYPSSKCTGTAE 119
SP|P0CAS6|PA2BF_CRODU WDIYRYSLKSGYITCGKGTWCKEQICECDRVAAECLRRSLSTYKNGYMFYPDSRCRGPSE 120
SP|Q1ZY03|PA2B_DEIAC  WDIYPYSWKTGVIICGEGTPCEKEICECDRAAAVCLGENLRTYKTKYMFYPDFLCKKPSK 120
SP|P04417|PA2B_GLOBL  WDDYTYSWKNGDIVCGGDDPCKKEICECDRAAAICFRDNLKTYKKRYMAYPDILCSSKSE 120
SP|A8CG84|PA2BS_DABSI SDRYKYKRVNGAIVCEKGTSCENRICECDKAAAICFRQNLNTYSKKYMLYPDFLCKGELR 120
SP|A8CG87|PA2A2_DABRR TATYSYSFENGDIVCGGDDPCLRAVCECDRVAAICFRENMNTYDKKYMLYSIFDCKEESD 120
                         * *     * * *     * . :****:.** *:  .  :*.      *    *

SP|P00626|PA2BA_VIPAA KC 122
SP|P86169|PA2BA_CRODR TC 122
SP|P11407|PA2BC_VIPAA KC 122
SP|P14424|PA2BB_VIPAA KC 122
SP|P0CAS5|PA2BE_CRODU TC 122
SP|P14421|PA2N_GLOHA  EC 122
SP|P00622|PA2B_BITCA  KC 121
SP|P0CAS6|PA2BF_CRODU TC 122
SP|Q1ZY03|PA2B_DEIAC  QC 122
SP|P04417|PA2B_GLOBL  KC 122
SP|A8CG84|PA2BS_DABSI C- 121
SP|A8CG87|PA2A2_DABRR QC 122
```

PLA2G2A, 5 reviewed sequences (the sequence of rabbit PLA2G2A has been discarded because incomplete**)**

```
CLUSTAL O(1.2.4) multiple sequence alignment
```

```
SP|P14555|PA2GA_HUMAN NLVNFHRMIKLTTGKEAALSYGFYGCHCGVGGRGSPKDATDRCCVTHDCCYKRLEKRGCG 60
SP|P14423|PA2GA_RAT   SLLEFGQMILFKTGKRADVSYGFYGCHCGVGGRGSPKDATDWCCVTHDCCYNRLEKRGCG 60
SP|P31482|PA2GA_MOUSE NIAQFGEMIRLKTGKRAELSYAFYGCHCGLGGKGSPKDATDRCCVTHDCCYKSLEKSGCG 60
SP|Q56JZ2|PA2GA_BOVIN DLLNFRKMIKLTTGKEPATRYSFYGCYCGMSGRGTPKDATDWCCRAHDCCYKNLESRGCR 60
SP|P47711|PA2GA_CAVPO HLKQFTEMIKLTTGKNGLTSYGAYGCHCGVGGKGTPKDATDRCCVRHDCCYDRLMKRGCG 60
                       : :* .** :.***.    *. ***:**:.*:*:****** **  *****. * . **

SP|P14555|PA2GA_HUMAN TKFLSYKFSNSGSRITCA-KQDSCRSQLCECDKAAATCFARNKTTYNKKYQYYSNKHCRG 119
SP|P14423|PA2GA_RAT   TKFLTYKFSYRGGQISCSTNQDSCRKQLCQCDKAAAECFARNKKSYSLKYQFYPNKFCKG 120
SP|P31482|PA2GA_MOUSE TKLLKYKYSHQGGQITCSANQNSCQKRLCQCDKAAAECFARNKKTYSLKYQFYPNMFCKG 120
SP|Q56JZ2|PA2GA_BOVIN TKFLKYNVTYQEDQIVCE-DADDCKSQVCQCDKIAANCFAANLKTYNKKLRFYNKFRCRG 119
SP|P47711|PA2GA_CAVPO TKFLNYRFTHKGSSITCSVKQNSCQKQLCECDKAAAYCFAANLKSYSRRYQFYYNGLCRG 120
                      **:*.*. :   . * *  . :.*:.::*:*** ** *** * .:*. : ::* :  *:*

SP|P14555|PA2GA_HUMAN STPRC 124
SP|P14423|PA2GA_RAT   KTPSC 125
SP|P31482|PA2GA_MOUSE KKPKC 125
SP|Q56JZ2|PA2GA_BOVIN AAPAC 124
SP|P47711|PA2GA_CAVPO KTPSC 125
                        * *
```

PLA2G2A 5 reviewed sequences and 5 unreviewed sequences

```
CLUSTAL O(1.2.4) multiple sequence alignment

SP|P14555|PA2GA_HUMAN          NLVNFHRMIKLTTGKEAALSYGFYGCHCGVGGRGSPKDATDRCCVTHDCCYKRLEKRGCG 60
SP|P14423|PA2GA_RAT            SLLEFGQMILFKTGKRADVSYGFYGCHCGVGGRGSPKDATDWCCVTHDCCYNRLEKRGCG 60
SP|P31482|PA2GA_MOUSE          NIAQFGEMIRLKTGKRAELSYAFYGCHCGLGGKGSPKDATDRCCVTHDCCYKSLEKSGCG 60
SP|Q56JZ2|PA2GA_BOVIN          DLLNFRKMIKLTTGKEPATRYSFYGCYCGMSGRGTPKDATDWCCRAHDCCYKNLESRGCR 60
SP|P47711|PA2GA_CAVPO          HLKQFTEMIKLTTGKNGLTSYGAYGCHCGVGGKGTPKDATDRCCVRHDCCYDRLMKRGCG 60
TR|A0A5K1UTF7|A0A5K1UTF7_HORSE HLLDFRKMIRLMTGKEATSSYGFYGCHCGVGGKGSPKDATDWCCVAHDCCYYRLQKRGCG 60
TR|H2PY77|H2PY77_PANTR         DLVNFHRMIKLTTGKEAALSYGFYGCHCGVGGKGSPKDATDRCCVTHDCCYKRLEKRGCG 60
TR|G3QZ00|G3QZ00_GORGO         DLVNFRRMIKLTTGKEAALSYGFYGCHCGVGGKGSPKDATDRCCVTHDCCYKRLEKRGCG 60
TR|A0A452FN06|A0A452FN06_CAPHI SLLDFRKMIKFATGKEPATNYSFYGCYCGMRGRGTPKDATDRCCRAHECCYRSLESRGCR 60
TR|A0A2R8ZCM9|A0A2R8ZCM9_PANPA DLVNFHRMIKLTTGKEAALSYGFYGCHCGVGGKGSPKDATDRCCVTHDCCYKRLEKRGCG 60
                                : :* .** : ***.    *. ***:**: *:*:****** **  *:***  * . **

SP|P14555|PA2GA_HUMAN          TKFLSYKFSNSGSRITCA-KQDSCRSQLCECDKAAATCFARNKTTYNKKYQYYSNKHCRG 119
SP|P14423|PA2GA_RAT            TKFLTYKFSYRGGQISCSTNQDSCRKQLCQCDKAAAECFARNKKSYSLKYQFYPNKFCKG 120
SP|P31482|PA2GA_MOUSE          TKLLKYKYSHQGGQITCSANQNSCQKRLCQCDKAAAECFARNKKTYSLKYQFYPNMFCKG 120
SP|Q56JZ2|PA2GA_BOVIN          TKFLKYNVTYQEDQIVCE-DADDCKSQVCQCDKIAANCFAANLKTYNKKLRFYNKFRCRG 119
SP|P47711|PA2GA_CAVPO          TKFLNYRFTHKGSSITCSVKQNSCQKQLCECDKAAAYCFAANLKSYSRRYQFYYNGLCRG 120
TR|A0A5K1UTF7|A0A5K1UTF7_HORSE TKLLNYKFSYRGGKIICA-KQDSCRSELCQCDKTAASCFARNRKTYSKKYQYYNNKSCRG 119
TR|H2PY77|H2PY77_PANTR         TKFLSYKFSNAGSRITCA-KQDSCRSQLCECDKAAANCFARNKTTYNKKYQYYSNKHCRG 119
TR|G3QZ00|G3QZ00_GORGO         TKFLSYKFSNVGSRITCA-KQDSCRSQLCECDKAAANCFARNKTTYNKKYQYYSNKHCRG 119
TR|A0A452FN06|A0A452FN06_CAPHI TKFLKYNATYEEDQIICE-DTDDCKSQVCQCDKIAASCFATNLKTYNKKLRFYNKLRCRG 119
TR|A0A2R8ZCM9|A0A2R8ZCM9_PANPA TKFLSYKFSNAGSRITCA-KQDSCRSQLCECDKAAANCFARNKTTYNKKYQYYSNKHCRG 119
                               **:*.*. :   . * *  . :.*:..:*:*** ** *** * .:*. : ::* :  *:*

SP|P14555|PA2GA_HUMAN          STPRC 124
SP|P14423|PA2GA_RAT            KTPSC 125
SP|P31482|PA2GA_MOUSE          KKPKC 125
SP|Q56JZ2|PA2GA_BOVIN          AAPAC 124
SP|P47711|PA2GA_CAVPO          KTPSC 125
TR|A0A5K1UTF7|A0A5K1UTF7_HORSE KTPRC 124
TR|H2PY77|H2PY77_PANTR         STPRC 124
TR|G3QZ00|G3QZ00_GORGO         STPRC 124
TR|A0A452FN06|A0A452FN06_CAPHI PAPQC 124
TR|A0A2R8ZCM9|A0A2R8ZCM9_PANPA STPRC 124
                                 * *
```
